# Hunter-gatherer admixture facilitated natural selection in Neolithic European farmers

**DOI:** 10.1016/j.cub.2023.02.049

**Published:** 2023-03-23

**Authors:** Tom Davy, Dan Ju, Iain Mathieson, Pontus Skoglund

**Affiliations:** 1 Ancient Genomics Laboratory, Francis Crick Institute, 1 Midland Road, NW1 1AT London, UK; 2 Department of Genetics, Perelman School of Medicine, University of Pennsylvania, 415 Curie Blvd, Philadelphia, PA 19104, USA; 3 Senior authors; 4 Twitter: @TomDavy_; 5 Lead contact

## Abstract

Ancient DNA has revealed multiple episodes of admixture in human prehistory during geographic expansions associated with cultural innovations. One important example is the expansion of Neolithic agricultural groups out of the Near East into Europe and their consequent admixture with Mesolithic hunter-gatherers.^1–4^ Ancient genomes from this period provide an opportunity to study the role of admixture in providing new genetic variation for selection to act upon, and also to identify genomic regions that resisted hunter-gatherer introgression and may thus have contributed to agricultural adaptations. We used genome-wide DNA from 677 individuals spanning Mesolithic and Neolithic Europe to infer ancestry deviations in the genomes of admixed individuals and to test for natural selection after admixture by testing for deviations from a genome-wide null distribution. We find that the region around the pigmentation-associated gene *SLC24A5* shows the greatest overrepresentation of Neolithic local ancestry in the genome (|Z| = 3.46). In contrast, we find the greatest overrepresentation of Mesolithic ancestry across the major histocompatibility complex (MHC; |Z| = 4.21), a major immunity locus, which also shows allele frequency deviations indicative of selection following admixture (p = 1 × 10^−56^). This could reflect negative frequency-dependent selection on MHC alleles common in Neolithic populations or that Mesolithic alleles were positively selected for and facilitated adaptation in Neolithic populations to pathogens or other environmental factors. Our study extends previous results that highlight immune function and pigmentation as targets of adaptation in more recent populations to selection processes in the Stone Age.

## In brief

Davy et al. study natural selection after admixture between hunter-gatherer and Neolithic farmer-associated individuals in ancient Europe. In later populations, they find an excess of Early Neolithic ancestry at a pigmentation-associated gene and of Mesolithic ancestry at the MHC locus, suggesting adaptive admixture.

## RESULTS AND DISCUSSION

Despite the evidence from studies of ancient DNA that admixture among Holocene populations is ubiquitous, less is known about how admixture provided variation for natural selection to act upon during transitional periods. Given that Holocene admixture was often associated with dramatic migrations or changes in lifestyle, we might expect an important role for adaptive introgression. Perhaps the best-studied example of ancient admixture is in the Mesolithic-Neolithic transition in Europe. As Early Neolithic groups expanded across Europe from Anatolia in the period from 10,000 to 5,000 years ago,^1–3,5–8^ they admixed with local Mesolithic hunter-gatherers, and by 6,000 years ago derived 20%–30% of their ancestry from these local groups.^1,4,5,9^ The admixed Neolithic ancestry thus found itself in a new cultural and geographical landscape, with a hypothesized increase in infectious disease load due to population density and proximity to domestic animals.^10^

Previous studies of natural selection in the European Neolithic have either compared allele frequencies or haplotype structure with other ancient and modern populations.^5,11–14^ However, no study to date has specifically attempted to identify adaptive admixture, despite the fact that this mode of adaptation has been repeatedly observed in humans. A recent study identified two optimal approaches to detect adaptive admixture in data from present-day populations, based on allele frequencies and local ancestry.^15^ Here, we adapt these two approaches to ancient populations with a new framework to obtain p values from genome-wide null distributions, investigating adaptive admixture in 677 Mesolithic and Early and Middle Neolithic (admixed Neolithic) individuals.

**Figure 1.**
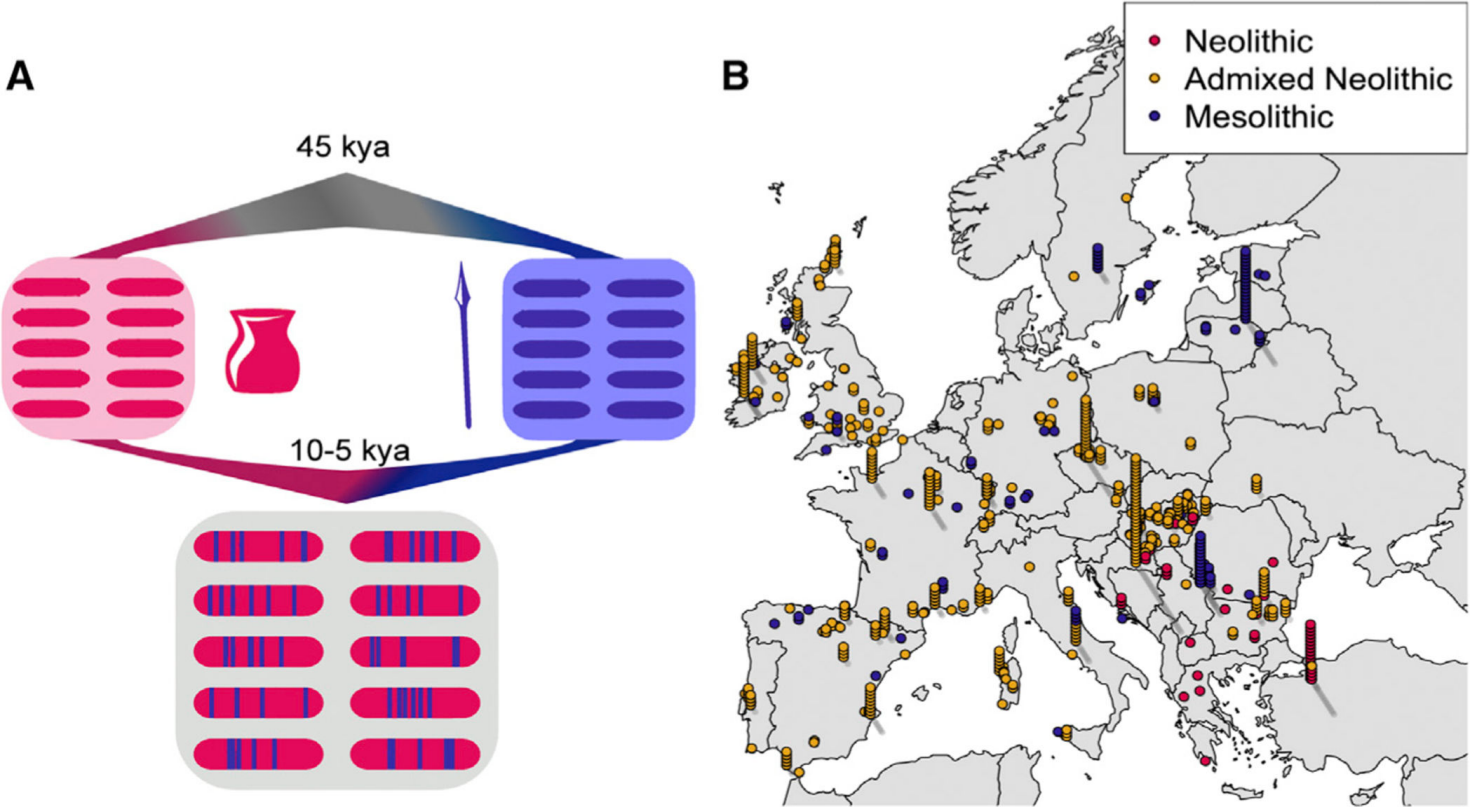
Admixture model and geographic distribution of Neolithic and Mesolithic individuals with genome-wide ancient DNA (A) An illustration of the genetic history of the Neolithic-Mesolithic transition in Western Eurasia. (B) ‘‘Casino-plot’’ of individuals included for analyses, colored by the ancestry group for which those individuals were used in this paper. For sites with multiple samples, we stack those individuals above the reported coordinates. See also Figure S1.

We clustered individuals with genome-wide ancient DNA data from Europe and Anatolia^1,2,4,5,9,16–39^ in the past ~15,000 years and assigned them to one of three groups: 125 Mesolithic and Upper Palaeolithic hunter-gatherer ancestry individuals, 55 Early Neolithic individuals from Anatolia and the Balkans without evidence for substantial hunter-gatherer admixture, and later admixed Neolithic individuals with substantial Mesolithic admixture. In total, our analysis contains 677 ancient individuals, spanning 7,500 years across West Eurasia (Figures 1A, 1B, and S1).

We first used an approach to find natural selection that was admixture-unaware, searching for increased differentiation as the squared allele frequency difference between populations, the *f*_2_ statistic.^40^ We computed the average for each SNP and 25 SNPs flanking SNPs on each side (i.e. in 51 base pair [bp] sliding windows), and obtain p values by fitting a gamma distribution to a null sample of 532 approximately independent loci, separated by at least 5 million base pairs (Mb). We observed no statistically significant outliers in the Mesolithic-Neolithic or Mesolithic-admixed Neolithic contrasts (Figure S2), which is likely due to the relatively deep divergence between these groups, i.e., variance in allele frequencies resulting from genetic drift obscuring signals of loci that are differentiated due to natural selection. However, in the Neolithic-admixed Neolithic contrast we observe a highly significant excess differentiation across the MHC region on chromosome 6, centered upon *HLA-DQB1* (p = 1 × 10^−21^). This suggests natural selection at the MHC in the period covered by the data from Neolithic and admixed-Neolithic groups, although this analysis does not address whether that might be due to adaptive Mesolithic admixture.

**Figure 2.**
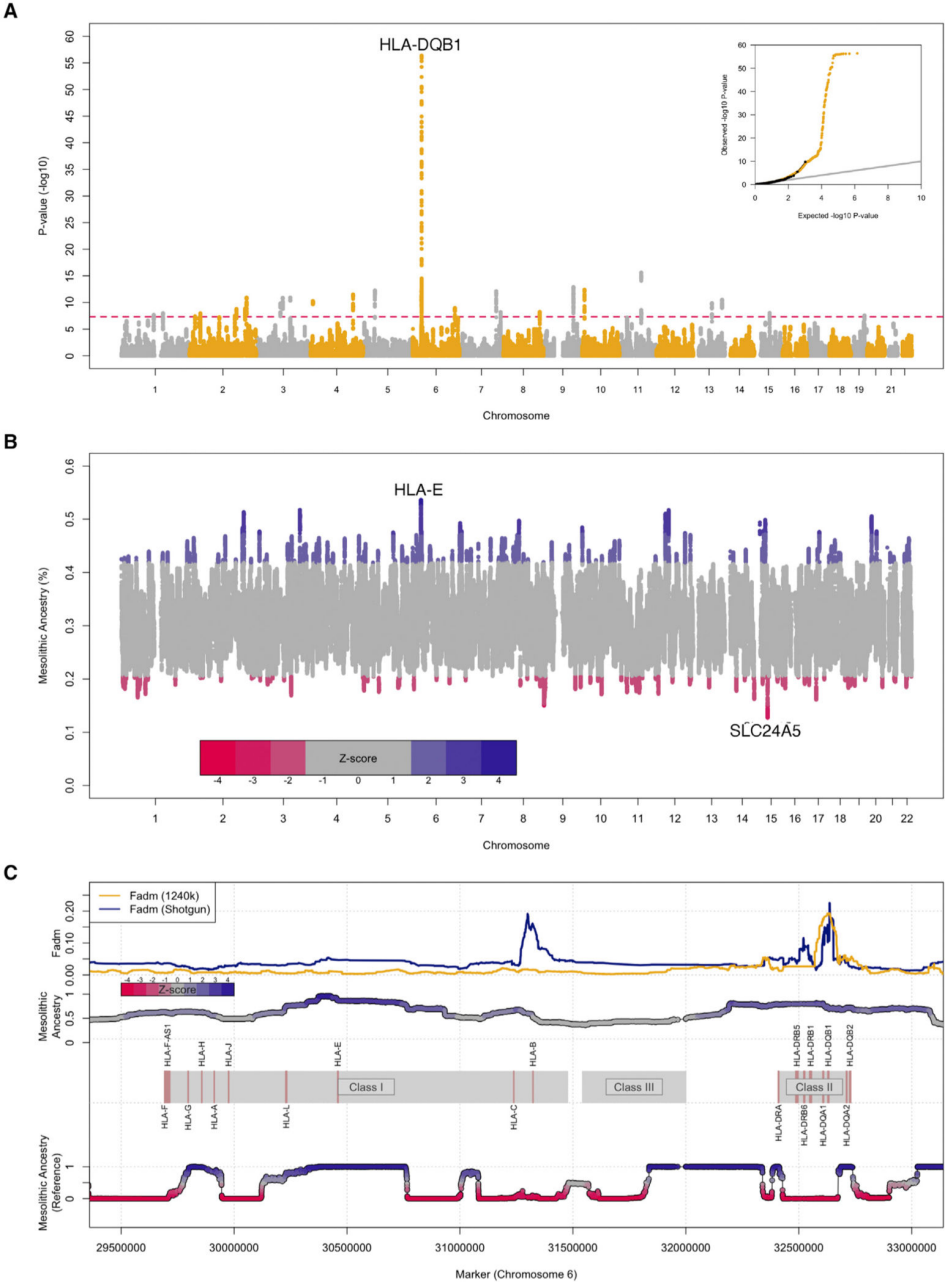
Genome-wide significant signals of adaptive admixture (A) Manhattan plot of p values from the *F*_adm_ scan across the genome for deviations from expected admixed allele frequencies. Inset, quantile-quantile plot of expected and observed p values. P values were obtained by fitting a gamma distribution to a null sample of 532 approximately independent loci, separated by at least 5 million base pairs (Mb). (B) Local ancestry deviations (LAD) in the Middle Neolithic across the genome, with top peaks of each ancestry labeled. (C) Zoomed-in region of the MHC (chromosome 6), with statistics derived from 1240k and whole-genome shotgun data across the MHC regions I, II, and III on chromosome 6.

To search for adaptive admixture, we applied a statistic based on previous work^5^ termed *F*_adm_,^15^ which tests for deviations from the expected allele frequencies given the genome-wide average mixture proportions of the contributing ancestries. We again observe an excess signal across the MHC, centered upon *HLA-DQB1* (p = 1 × 10^−56^) (Figure 2A). To confirm that our findings were not driven by ascertainment bias in the 1.2 million SNP panel,^41^ we analyzed the MHC region in 173 whole-genome shotgun sequences from 65 Mesolithic, 25 Neolithic, and 83 admixed Neolithic individuals^4,5,8,9,17,18,25–27,30,33,39,42–45^ (STAR Methods, Table S2) and observed a peak concordant to the 1240k results in both position and summary statistic stretching across the class II MHC region (Figure 2C).

We next sought to quantify the direction of admixture by searching for deviations in local ancestry across the genome (local ancestry deviation [LAD]^15^). We inferred local ancestry in 537 admixed Middle Neolithic individuals with genome-wide SNP data using *ancestryHMM,*^46^ which estimates local ancestry in low-coverage genomic data using allele frequencies from two populations. We computed standard errors and *Z* scores for LAD using an approximately independent subsample of the genome-wide distribution consisting of 555 sites separated by at least 5 Mb (STAR Methods).

The greatest excess of Neolithic ancestry centered on *SLC24A5* (Figures 2D and S3C), with a peak of +17.82% (|Z| = 3.46). The derived *SLC24A5* allele, which is carried on the Neolithic ancestry background, is one of the two alleles which contributes most to light skin pigmentation in present-day West Eurasian-ancestry populations.^47^ It has previously been shown to have been at relatively high frequency in the Neolithic population and absent in the Mesolithic hunter-gatherers,^5^ and our results show that the selection removed hunter-gatherer ancestry at this locus in later admixed Neolithic groups.

Meanwhile, the lowest amount of Neolithic ancestry is found at the MHC region on chromosome 6. Within this locus, the region of highest Mesolithic ancestry is centered on *HLA-E*, with a peak excess of +23.1% (|Z| = 4.21), and a secondary peak centered upon the class II region of +17.18% (|Z| = 3.10). This region of elevated Mesolithic ancestry continues as a contiguous region which extends across the class II region of the MHC, with an average of ancestry across the entire MHC (between positions 28,477,797–33,448,354 of chromosome 6) of +9.16% (|Z| = 1.60). (Figure 2C). Bias in LAD analysis at the HLA locus has been suggested to be expected in the direction of the source ancestry with higher diversity,^48^ unlike what we observe here. We also run *ancestryHMM* on the human reference genome to test the hypothesis of biased mapping but find that the reference is inferred to have both Neolithic and Mesolithic ancestry at this locus (Figure 2C). *AncestryHMM* and other local ancestry approaches have not been extensively used with ancient genetic data and may be sensitive to particular properties of these data. However, the LAD results are largely concordant with the *F*_adm_ results, suggesting that the approach is robust.

**Figure 3.**
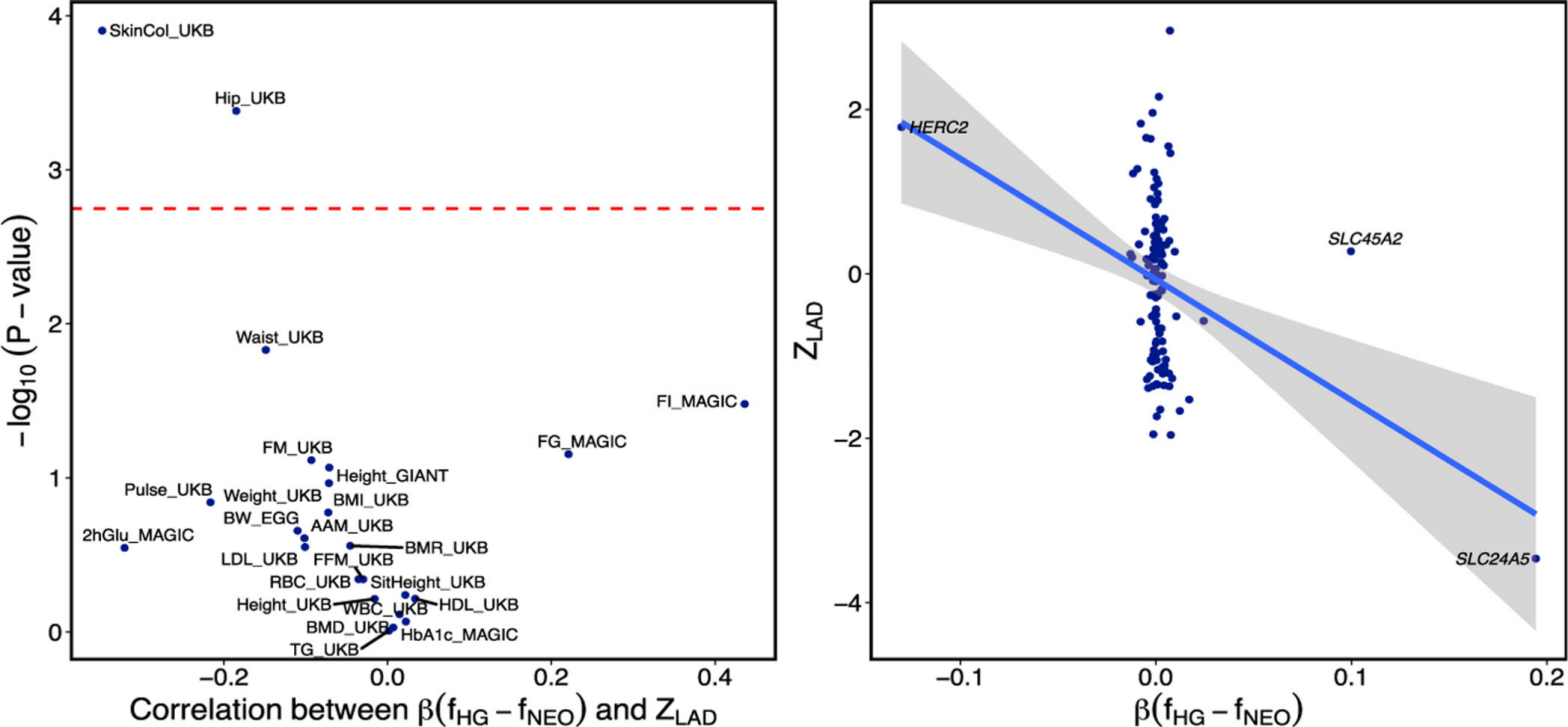
Test for polygenic adaptive admixture (A) Pearson correlation (p values) of polygenic traits against local ancestry. (B) Correlation of LAD *Z* scores with skin color SNP effect size weighted by the signed allele frequency difference between the two source populations.

It is also possible that adaptive admixture acted on multiple variants with small effect, spread across the genome. To test for evidence of such polygenic selection, we computed the Pearson correlation between the LAD and the implied local ancestry effect size for 38 traits in the UK BioBank, using genome-wide significant SNPs thinned to be approximately independent^49^ (Figure 3A). We see significant evidence of correlation between trait scores and LAD in skin color (p = 1.2 × 10^−4^), consistent with adaptive admixture around *SLC24A5*. Indeed, this signal is solely driven by two loci (Figure 3B), wherein a *HERC2* variant with a skew towards Mesolithic ancestry (Z = 1.8) also contributes to a lighter level of skin pigmentation alongside *SLC24A5*. Without these two loci, there is no significant evidence of polygenic selection (p = 0.42). We also observe a weaker but significant correlation for hip size (Figure 3A).

The Neolithic transition brought about drastic changes in demography, culture, and diet, as well exposure to novel pathogens and increased potential of zoonotic disease. In admixed Neolithic individuals, we found excess Neolithic farmer ancestry at the pigmentation locus *SLC24A5* and excess Mesolithic ancestry at the MHC immunity locus. Previous studies also found evidence of natural selection at *SLC24A5* in European populations^47,50^ and showed that the allele was almost fixed in Anatolian farmers and was introduced into Western Europe during the Neolithic^5,12,33,51^ but our study now further demonstrates that subsequent selection during the Middle Neolithic was rapid enough to result in the removal of Mesolithic ancestry across the wider locus, covering an approximately 3 Mb region. In a similar but opposite process, the MHC locus has previously been demonstrated to have undergone selection in the ancestors of present-day Europeans^5,52^ and specifically in Neolithic Europe.^11^ Here, we obtain further robust results for selection at the MHC locus corrected for multiple testing and demonstrate that this process specifically increased hunter-gatherer ancestry at the locus.

In contrast to *SLC24A5*, the second high-effect pigmentation variant in *HERC* displays an excess of Mesolithic ancestry (+10.79%, |Z| = 1.90). Together with the third high-effect pigmentation variant at *SLC45A2*, which arrived in Europe via later expansions from the steppe, selection on pigmentation in Europe thus targeted variants from each of the three major ancestral populations.^4^ This highlights the prominent role of admixture in the evolution of skin pigmentation in Western Eurasia. That this signal is not found in the allele frequency-based analysis with *F*_adm_ can likely be attributed to the small absolute change in allele frequency between our Neolithic populations, confirming recent demonstrations that local ancestry can in some cases be more powerful than allele frequency analysis for detecting selection in admixed populations.^15^

Evidence of selection on Mesolithic ancestry across the MHC locus highlights its role in facilitating adaptation in immunity during the Neolithic transition in Europe. One hypothesis is that this reflects the fact that Neolithic populations were expanding into environments containing pathogens to which Mesolithic populations had already adapted. This is contrary to the idea that the pathogen load in Neolithic populations was solely driven by increased population density and proximity to zoonotic vectors via animal husbandry. On the other hand, although examples of putative adaptive admixture involving the MHC have been previously described,^48,53,54^ no clear link between the alleles under selection within this region and a specific pathogen has been identified.

Another possibility is that this adaptation reflects negative frequency-dependent selection because pathogens adapt to the most common alleles in the population, making rare alleles beneficial.^55^ In particular, MHC class II genes have been suggested to experience a red-queen-like arms race between the binding ability of antigen-presenting proteins and pathogens’ ability to escape this binding and subsequent immune response.^56^ Under this model, HLA alleles unseen by a given pathogen will have higher fitness initially following admixture owing to rarity. Thus, selection on the minor Mesolithic ancestry could simply reflect admixture introducing rarer variants into Neolithic populations, increasing diversity at the locus. This can also be explained as selection for heterozygosity in class II genes to diversify the immunity within the population. One caveat to this interpretation is that we are pooling samples across large regions and time periods, and there is a possibility that our results reflect more localized differentiation or natural selection in regions and periods with more sampled diversity. On the other hand, we note that a similar effect has been observed in present-day populations; Cuadros-Espinoza et al.^15^ find adaptive admixture at the MHC, reporting, similarly, ~50% local ancestry for the minority admixing ancestry derived from west rainforest hunter-gatherers in agricultural Western Bantu speakers, echoing previous analyses.^53,57^

We note that the HLA appears to be a frequent target of natural selection and the processes described above are not exclusive. Extensive evidence of selection has been detected both in scans focusing on the past few millennia,^5,11,14,52,58^ and following introgression from archaic humans.^54,59^ Future studies, including whole-genome shotgun data in tandem with improved functional annotation, may shed further light on this adaptive process.

## STAR★METHODS

### RESOURCE AVAILABILITY

#### Lead contact

Requests for further information should be directed to and will be fulfilled by the lead contact, Pontus Skoglund (pontus.skoglund@crick.ac.uk)

#### Materials availability

This study did not generate new unique reagents.

#### Data and code availability

Any additional information required to reanalyze the data reported in this paper is available from the lead contact upon request. This paper analyses existing, publicly available data. These accession numbers for the datasets are listed in Tables S1 and S2. Code from this paper used to generate all figures and analyses from allele frequency data is available at https://github.com/Tom-Davy/mesoneo_admixture.

### EXPERIMENTAL MODEL AND SUBJECT DETAILS

No experimental models were used in the study.

### METHOD DETAILS

#### Data preparation

We first used clustering approaches on a large set of previously published^1–9,16,18,24,25,27,28,30,33,37,44,45,51,71–84^ individuals to identify those individuals exclusively or almost exclusively Hunter-Gatherers (Mesolithic, Western or Siberian Hunter-Gatherers [WHG, SHG]) or Neolithic ancestry (Early Neolithic, which we identify using Neolithic Anatolia as a baseline). We downloaded v50 of 10,391 individuals (3,589 ancient) from the Reich lab https://reich.hms.harvard.edu/downloadable-genotypes-present-day-and-ancient-dna-data-compiled-published-papers, accessed July 19^th^ 2022 for the 1240K set of SNPs. We ran a first set of clustering with ADMIXTURE from K = 3 to K = 11 on all ancient individuals, removing relatives and duplicates based on assignment as such in the Reich lab compendium. Given the choice, we select the highest coverage individual or family member. We then selected individuals clustering with known examples that typify Mesolithic or Neolithic ancestry, or were clearly admixed, at K = 5, dated to >5kya, excluding siblings, parents, and duplicates. We then further filtered on age, setting a max age for Mesolithic, Neolithic and Admixed Neolithic of 12kya, 8.5kya and 8kya respectively, retaining 677 individuals, which we recluster alone with 40 individuals labeled MSL to confirm ancestry proportions and classification of ancestry (Figure S1). Using PLINK, we then make a subset of files containing only individuals fitting into either Mesolithic (EHG + WHG) or admixed Neolithic ancestry at K = 3 in this cluster, alongside a geographically and temporally conservative Neolithic cluster. We then remove sites below 99% mappability as defined on the human reference genome (hs37d5).

#### Confirmation of HLA signal in whole-genome shotgun data

To confirm the presence of the HLA signal, we collected a set of 99 individuals (Table S2), including some used in the original analyses, which had whole-genome shotgun genomes, and added 74 published and unpublished genomes available pre-publication under the Ft. Lauderdale principles from the AGDP (https://reich.hms.harvard.edu/ancient-genome-diversity-project).

We compile all shotgun individuals using htslib with the pileup flag into randfa format, filtering for a base quality score of 20 and a mappability quality score of 30. We thus only use this data for estimating F_adm_ in a restricted region comprising the MHC region. We base our defined populations on the annotation in the 1240k Reich compendium v50.0. We filter the MHC region for SNPs with a minimum allele frequency higher than 0.05, and only retain the intersect between these SNPs and the hs37d5 mappability filter for sites > 0.99, retaining 21,007 sites using BEDTools.^67^

#### Testing the hypothesis of mapping bias in the MHC region

The MHC locus can be difficult to analyze, due to the high allelic diversity, especially across the class II genes. Hence, we sought to understand if there could be any underlying bias in the reference genome that could be driving signals here (Figure 2C). We examine pairwise *f*_2_ statistics between the reference genome and our ancient populations, alongside targeting the human reference genome in a LAD analysis under the same conditions as our initial local ancestry analysis. We see no overall bias in *f*_2_ at the locus where *F*_adm_ reports selection. Furthermore, we show that the reference genome contains both Mesolithic and Neolithic ancestries across the MHC, with our top two local ancestry signals in the locus contained within different ancestries within the reference genome.

### QUANTIFICATION AND STATISTICAL ANALYSIS

#### Detecting selective sweeps

We filtered the full set of SNPs by conditioning on observing at least 20 non-missing pseudohaploid genotypes in the Neolithic out of a maximum of 51. We apply a sliding window of 51 SNPs. We utilized the ${\text{F}}_{\text{adm}}$ statistic, i.e. ${\text{F}}_{\text{adm}}={{(\text{p}}_{\text{ADM}}-\text{y})}^{\text{2}}{/(2\;\text{x}(1\;-\text{y})}^{2}{-(1\;-\text{y}))}^{2}$, where ${\text{p}}_{\text{ADM}}$ is the frequency of a given SNP in the admixed population and y is an expectation of allele frequency derived by the contributing ancestry proportions (Anc_MESOLITHIC_ and Anc_NEOLITHIC_) weighted by allele frequency: ( p_MESOLITHIC_ * Anc_MESOLITHIC_ + p_NEOLITHIC_ * Anc_NEOLITHIC_). Anc_MESOLITHIC_ was set to 0.3 and Anc_NEOLITHIC_ to 0.7. In all cases, the given statistic is calculated on a per-SNP basis before a sliding-window is applied across the genome with a step size of 1. We derive the *f*_2_ analyses in an identical manner. We annotate genes using the gencode v39liftover37 annotation files,^60^ and denote them based on the centering of the window where the center SNP is the median value of the SNP window, which is always odd-numbered.

For each analysis, we then draw a null distribution from this sliding-window whole-genome distribution, ensuring that each sliding-window datum that contributes to the null distribution is sampled with an array of SNPs that contain no positions less than 5Mb away from the previous sample. A gamma distribution is then fitted to this null distribution using the R^61^ package *fitdistrplus v1.1–3*^62^ with the flags ““gamma”, method = “mle”, keepdata = T”’. We then derive p values for the genome-wide distribution from this null-fitted gamma distribution.

#### Detection of local ancestry outliers

To analyze biases in local ancestry, we bring the same panel of 677 individuals (Table S1) forward to analysis with *ancestryHMM*.^46^ We derive frequency counts for both the contributing ancestries (Mesolithic, Neolithic) and similarly obtain this for each individual admixed Neolithic sample via PLINK v1.9b.^63^ We then run *ancestry HMM* with a prior of 30% Mesolithic and 70% Neolithic ancestry, and an *N*_e_ of 10,000, which we take to be a fair assumption of Mid- to Late Neolithic effective population size,^84^ via the following command line: ancestry_hmm -i ../hmm.$c.input.tsv -s $hmm_input_file -a 2 0.3 0.7 -p 0 0 0.3 -p 1 35 0.7 –ne 10000. We derive *Z* scores first by taking a null distribution as in the $F_{\text{adm}}$ analysis of LA values and calculate *Z* scores for the genome-wide distribution using that distribution’s mean.

#### Detection for biased inheritance of polygenic trait alleles

As in Mathieson and Terhorst,^49^ we start with 28 quantitative traits of interest, subsetting SNPs with a GWAS p value of *P* < 1e^−8^ overlapping the 1240k SNP array. We then iteratively prune for each trait, subsetting the smallest p value and removing all other associations within 250Kb. Departing from Mathieson & Terhost, we weight these pruned effect sizes by the difference in allele frequency between Mesolithic and Neolithic, sum these across the genome for each trait and test for correlation to the LAD derived in the local ancestry analysis via a Pearson correlation test.

## Supplementary Material

MMC3

MMC2

MMC1

## Figures and Tables

**Table:** KEY RESOURCES TABLE

REAGENT or RESOURCE	SOURCE	IDENTIFIER

Deposited data		

Allen Ancient DNA Resource	Reich Lab	https://reich.hms.harvard.edu/allen-ancient-dna-resource-aadr-downloadable-genotypes-present-day-and-ancient-dna-data
Allen Ancient Genome Diversity Project	Reich Lab	https://reich.hms.harvard.edu/ancient-genome-diversity-project
Gencode v39liftover37 gene annotations	Frankish et al.^60^	https://genome.ucsc.edu/cgi-bin/hgTables?db=hg19&hgta_group=genes&hgta_track=wgEncodeGencodeV39lift37&hgta_table=wgEncodeGencodeCompV39lift37&hgta_doSchema=describe+table+schema
Human reference genome NCBI build 37, GRCh37	Genome Reference Consortium	http://www.ncbi.nlm.nih.gov/projects/genome/assembly/grc/human/

Software and algorithms		

R v4.1.0	Team, R.C.^61^	https://www.r-project.org/
Fitdistrplus v1.1–3	Delignette-Muller et al.^62^	https://CRAN.R-project.org/package=fitdistrplus
PLINKv1.9b	Chang et al.^63^	www.cog-genomics.org/plink/1.9/
AncestryHMM	Corbett-Detig and Nielsen^46^	https://github.com/russcd/Ancestry_HMM
Admixture v1.3.0	Alexander et al.^64^	https://dalexander.github.io/admixture/
Htslib	Bonfield et al.^65^	https://github.com/samtools/htslib
Popstats	Skoglund et al.^66^	https://github.com/pontussk/popstats
BEDTools	Quinlan and Hall^67^	https://bedtools.readthedocs.io/en/latest/
Plotrix	Lemon J.^68^	https://cran.r-project.org/web/packages/plotrix/index.html
Scales	Wickham and Seidel^69^	https://cran.r-project.org/web/packages/scales/index.html
Data.table	Dowle andSrinivasan^70^	https://cran.r-project.org/web/packages/data.table/index.html
Maps	N/A	https://cran.r-project.org/web/packages/maps/index.html
Original code used in this paper	This paper	https://github.com/Tom-Davy/mesoneo_admixture

## Highlights

- Genomic evidence for adaptive admixture in Stone Age Europe
- Excess of Neolithic farmer ancestry around the pigmentation-associated gene ***SLC24A5***
- Excess of hunter-gatherer ancestry in the major histocompatibility complex (**MHC**)
- Post-admixture selection occurred upon variants from both admixing ancestries
