# Supplementary material for: Hunter-gatherer admixture facilitated natural selection in Neolithic European farmers
Source: Curr Biol. Author manuscript; Available in PMC 2023 May 2. (PMC10153476; doi:10.1016/j.cub.2023.02.049)
Supplement: MMC1 [file NIHMS1882585-supplement-MMC1.pdf]

Current Biology, Volume *33*

Supplemental Information

Hunter-gatherer admixture facilitated natural selection in Neolithic European farmers

Tom Davy, Dan Ju, Iain Mathieson, and Pontus Skoglund

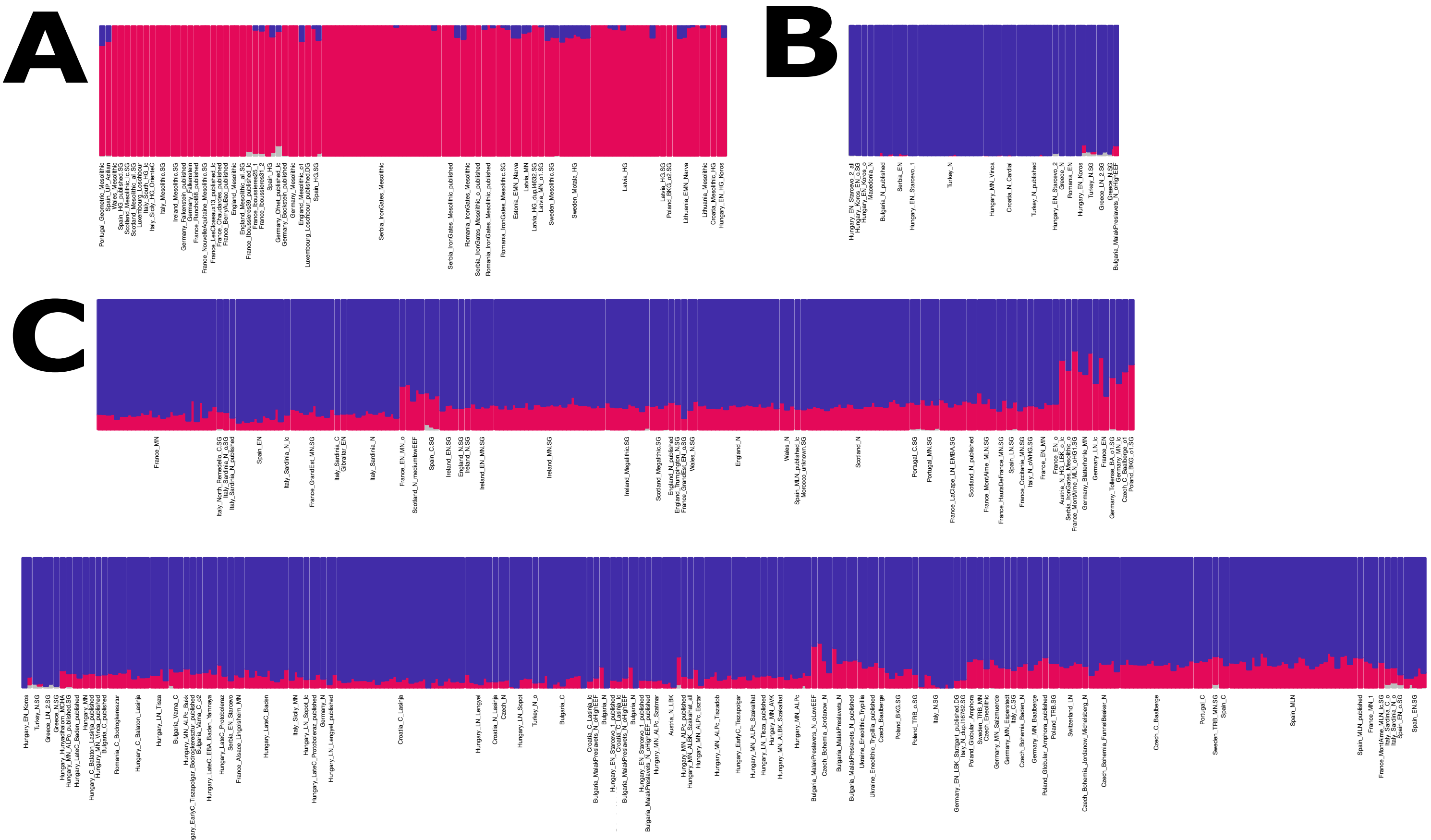

A
B
C

Figure S1. Clustering results of 677 Mesolithic, NEO, and MNEO individuals alongside 40 individuals from the MSL (Mende from Sierra Leone) 1000 genomes panel (the latter not shown) obtained using ADMIXTURE with K=3 (STAR Methods). Related to Figure 1. A) Mesolithic, B) Neolithic and C) Admixed Middle Neolithic.

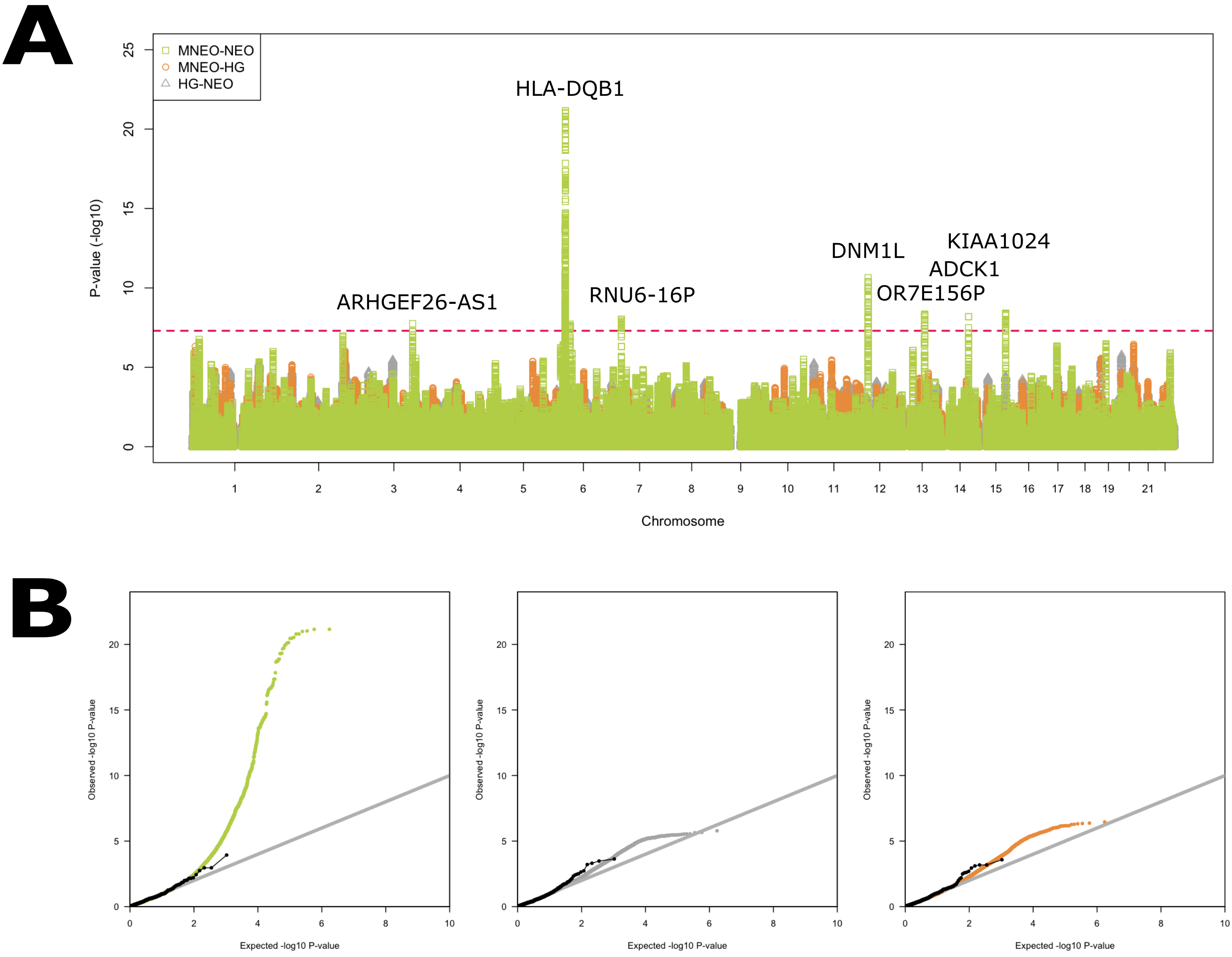
A
MNEO-NEO
MNEO-HG
HG-NEO
HLA-DQB1
ARHGEF26-AS1
RNU6-16P
DNM1L
KIAA1024
ADCK1
OR7E156P
P-value (-log10)
Chromosome
B
Observed -log10 P-value
Expected -log10 P-value

Figure S2. f_2 pairwise comparisons of ancient populations. Related to STAR methods. A) Manhattan plot of p-values for each pairwise comparison. B) Quantile-quantile plots for each pairwise comparison.

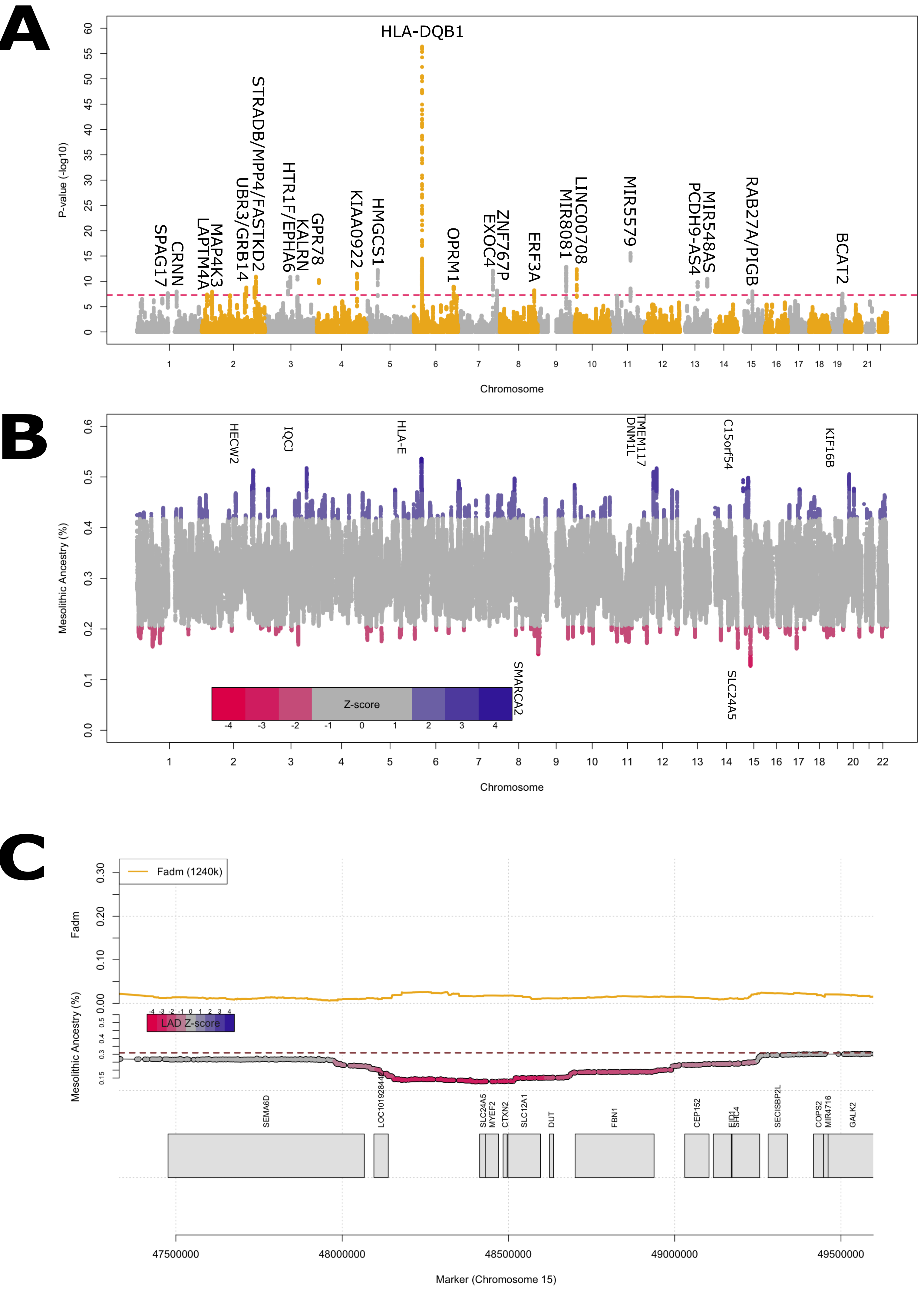
A
B
C
HLA-DQB1
SPAG17
CRNN
LAPTM4A
MAP4K3
UBR3/GRB14
STRADB/MPP4/FASTKD2
HTR1F/EPHA6
KALRN
GPR78
KIAA0922
HMGCS1
OPRM1
EXOC4
ZNF767P
ERF3A
MIR8081
LINC00708
MIR5579
PCDH9-AS4
MIR548AS
RAB27A/PIGB
BCAT2
P-value (-log10)
Chromosome
HECW2
IQCJ
HLA-E
DNM1L
TMEM117
C15orf54
KIF16B
SMARCA2
SLC24A5
Z-score
Mesolithic Ancestry (%)
Fadm (1240k)
Fadm
LAD Z-score
SEMA6D
SLC24A5
MYEF2
CTXN2
SLC12A1
DUT
FBN1
CEP152
SECISBP2L
COPS2
MIR4716
GALK2
Marker (Chromosome 15)

Figure S3. Fully annotated versions of adaptive admixture test manhattan plots as seen in Figure 2 alongside a zoomed-in region of chromosome 15. Related to Figure 2B. A) F_{adm} B) Local Ancestry Deviation.
C) Adaptive admixture statistic derived from 1240k data alongside local ancestry signal at the SLC24A5 locus on chromosome 15.
